# Relationship between moderate-to-vigorous, light intensity physical activity and sedentary behavior in a prospective cohort of older French adults: a 18-year follow-up of mortality and cardiovascular events ─ the PROOF cohort study

**DOI:** 10.3389/fpubh.2023.1182552

**Published:** 2023-06-07

**Authors:** Caroline Dupré, Marlène Brégère, Mathieu Berger, Vincent Pichot, Sébastien Celle, Martin Garet, Hervé Fundenberger, Nathalie Barth, Jessica Guyot, Bienvenu Bongue, Jean-Claude Barthélémy, Frédéric Roche, David Hupin

**Affiliations:** ^1^Jean Monnet University Saint-Etienne, Mines Saint-Etienne, University Hospital of Saint-Etienne, INSERM, U1059, DVH Team, SAINBIOSE, Saint-Étienne, France; ^2^Jean Monnet University Saint-Etienne, University Hospital of Saint-Etienne, Presage Institute, Chaire Santé des Ainés, Saint-Étienne, France; ^3^University Hospital of Saint-Etienne, Faculty of Medicine, Jean Monnet University, Saint-Étienne, France; ^4^Department of Clinical and Exercise Physiology, University Hospital Center, Saint-Étienne, France; ^5^Gérontopôle Auvergne Rhône-Alpes, Saint-Étienne, France; ^6^Centre Technique d’Appui et de Formation des Centres d’Examens de Santé, Saint-Étienne, France

**Keywords:** physical activity, sedentary behavior, mortality risk, cardiovascular events, cohort study

## Abstract

**Background:**

It is well documented that moderate-to-vigorous intensity physical activity (MVPA) is effective in the prevention of major chronic diseases. Even though the current international physical activity (PA) guidelines still mainly focus on MVPA, the topic of the most recent epidemiological studies has shifted from MVPA to light intensity physical activity (LPA), owing to the necessity of promoting all activities vs. sedentary behavior (SB). However, the evidence remains currently limited. Thus, the clarification of the effects of LPA and the close relationship with SB is crucial to promote public health.

**Method:**

PA and SB were assessed by a validated self-administered questionnaire (POPAQ) investigating 5 different types of PA during the 7 previous days. PA was measured in metabolic equivalent of task (MET)-h, which refers to the amount of energy (calories) expended per hour of PA. SB was measured in hour/day. Medical histories and examinations were taken during each clinical visit to determine clinical events. All-cause mortality was established using the same procedure and by checking local death registries. The relationships between the intensity of PA (light, moderate to vigorous) and mortality and between the periods of SB and mortality or CV events were analyzed by splines and COX models, adjusted for sex and year of birth.

**Results:**

From the 1011 65-year-old subjects initially included in 2001 (60% women), the last 18-year follow-up has been currently completed since 2019. A total of 197 deaths (19.2%, including 77 CV deaths) and 195 CV events (19.3%) were reported. Averages (standard deviation) of MVPA, LPA and SB were, respectively, 1.2 h/d (0.3), 5.8 h/d (1.1), and 6.6 h/d (2.3). For all-cause deaths, as well as CV deaths, the splines were significant for LPA (*p* = 0.04 and *p* = 0.01), and MVPA (*p* < 0.001 and *p* < 0.001), but not for SB (*p* = 0.24 and *p* = 0.90). There was a significant reduction in CV events when SB was decreasing from 10.9 to 3.3 h/d.

**Conclusion:**

The PROOF cohort study shows a clear dose–response between the dose of LPA, MVPA, SB and risk of mortality. These findings provide additional evidence to support the inclusion of LPA in future PA guidelines.

## Highlights

- An equivalent daily dose of walking at low intensity (at least 1.5 h at one’s own pace) has already a beneficial effect on health in older adults with a 30% reduction in mortality risk.
- This reduction in mortality risk is even more strong (80%) from 3 h of walking at low intensity per day.
- These results are even stronger in those who have chronic conditions (hypertension or type 2 diabetes).

## Background

It is well documented that moderate-to-vigorous intensity physical activity (MVPA) is effective in the prevention of major chronic disease, including cardiovascular (CV) diseases (1–3). Previous World Health Organization (WHO) physical activity (PA) guidelines recommended that adults engage in at least 150 min of MVPA in a week in bouts of at least 10 min to achieve health benefits (4). Even though the current international PA guidelines still mainly focus on MVPA, these are now accounted for from the first minutes (5–7). Also, the topic of the most recent epidemiological studies has shifted from MVPA to light intensity physical activity (LPA), owing to the necessity of promoting all activities and not only those which are moderate or vigorous intensity (sport) vs. sedentary behavior (SB) (8). Sitting time or SB and LPA, mainly activities of everyday living in occidental countries during this 21st century, account for a major part of total daily activities for average older people (9). Indeed, only a small period of the day is spent in MVPA. Even though higher intensities of PA lead to generally better health outcomes, LPA may be more accessible than MVPA for older adults or people with chronic diseases. Even if SB is truly independent of PA, it has been suggested that high doses of PA (whatever intensities) could attenuate the risks of prolonged sitting (10). Consequently, health promotion programs and public health guidelines should emphasize the need for the public to engage in PA with high priority consideration to PA of lower intensity. However, the evidence remains currently limited. Thus, the clarification of the effects of LPA and the close relationship with SB is crucial to promote public health.

The aim of this study was therefore to investigate the associations of assessed MVPA, LPA and SB with all-cause mortality and mortality from cardiovascular disease (CVD) in a French population-based cohort with 18 years follow-up time.

## Method

The PROOF (PROgnostic indicator OF cardiovascular and cerebrovascular events) cohort study was designed to prospectively assess the predictive value of CV risk factors and autonomic nervous system activity level among a healthy retired French population, regarding CV events and mortality (all-cause, CVD) (11). PA was assessed by the population physical activity questionnaire (POPAQ), investigating 5 different types of PA (domestic and work-related activities, transportation, leisure time and sports) during the 7 previous days (12–14). This self-administered questionnaire, validated against maximal oxygen consumption, is designed to provide a complete picture of a subject’s usual PA (15). PA was measured in metabolic equivalent of task (MET)-h, which refers to the amount of energy (calories) expended per hour of PA. Based on Ainsworth’s compendium of PA,^1^ resting energy expenditure is assumed to be 1 metabolic unit (MET). PA of 1.6–2.9 METs (including casual walking, doing household shores or activities of daily living) is defined as light, 3–5.9 METs (including brisk walking) is considered as moderate, and PA ≥ 6 METs (including sports activities) as vigorous. All periods in sitting time < 1.5 METs are recognized as SB (16, 17). For example, 1.5 h of 2 METs PA (light intensity) 5 d/week was equivalent to 15 METs-h/week of LPA. A combination of 4 METs PA (moderate intensity) for 1 h and 6 METs PA (vigorous intensity) for 0.5 h 3 d/week is equivalent to 21 METs-h/week of MVPA. Medical histories (CV risk factors notably) and treatments (CV drug therapies), anthropometric measures (body mass index, BMI), clinical (blood pressure) and biological [glycemia, lipid profile and C-reactive protein (CRP)] examinations were taken during each clinical visit to the research center to determine clinical events, and missing information were obtained from reviews of hospital charts and questionnaires sent to family practitioners. All-cause mortality was established using the same procedure and by checking local death registries for every missed medical examination. Death certificates were individually analyzed. In addition, new onset of risk factors or events such as hypertension, diabetes and CV events were checked for and updated at each clinical visit. Late fatal or nonfatal events were continued to be monitored after the examination programs. Subjects who had not participated in a survey (because of dropouts or loss of autonomy) were excluded from the study. Dropouts could return at a later survey if they changed their mind since it concerned those who definitively did not wish to continue the study. The loss of autonomy could justify institutionalization and therefore the subjects were definitively excluded. Data were collected on paper forms and given to an independent company to ensure a double-blind data capture. The resulting database was analyzed by an independent statistician to detect outliers and inconsistencies. Discrepancies were then checked using the subject forms by a medical doctor to ensure statistical and medical coherence. The baseline characteristics of the subjects at baseline were compared according to the occurrence of events or not. The relationship was tested by χ^2^ and Wilcoxon tests when appropriate. The relationships between the dose of PA (light, moderate to vigorous) and mortality and between the periods of SB and mortality or CV events were analyzed by splines and COX models, adjusted for sex and year of birth. The use of splines made it possible to model the relationship between PA and mortality/CV events as well as to compensate for the possible nonlog-linearity of the relationship. Several nodes were tested, and the choice was made by minimizing the Akaike information criterion. The knots obtained on the splines between PA and mortality/ CV events, the dose of PA and SB were transformed into new classes: ]0–6.2[, [6.2–14.5[, ≥14.5 MET-h/week for MVPA, [0–3[,]3–5.5[and ≥ 5.2 h/d for LPA and [0–3.3[,]3.3–6.5[, [6.5–10.9[, ≥10.9 h/d for SB. These classes were then introduced into COX models, adjusted for sex and year of birth in a first model. A second model was additionally adjusted for comorbidities (family history, hypertension, dyslipidemia, type 2 diabetes and smoking, triglycerides and CRP). The reference category was a low dose MVPA or LPA, and strong sedentary lifestyle.

## Results

**Figure 1:**
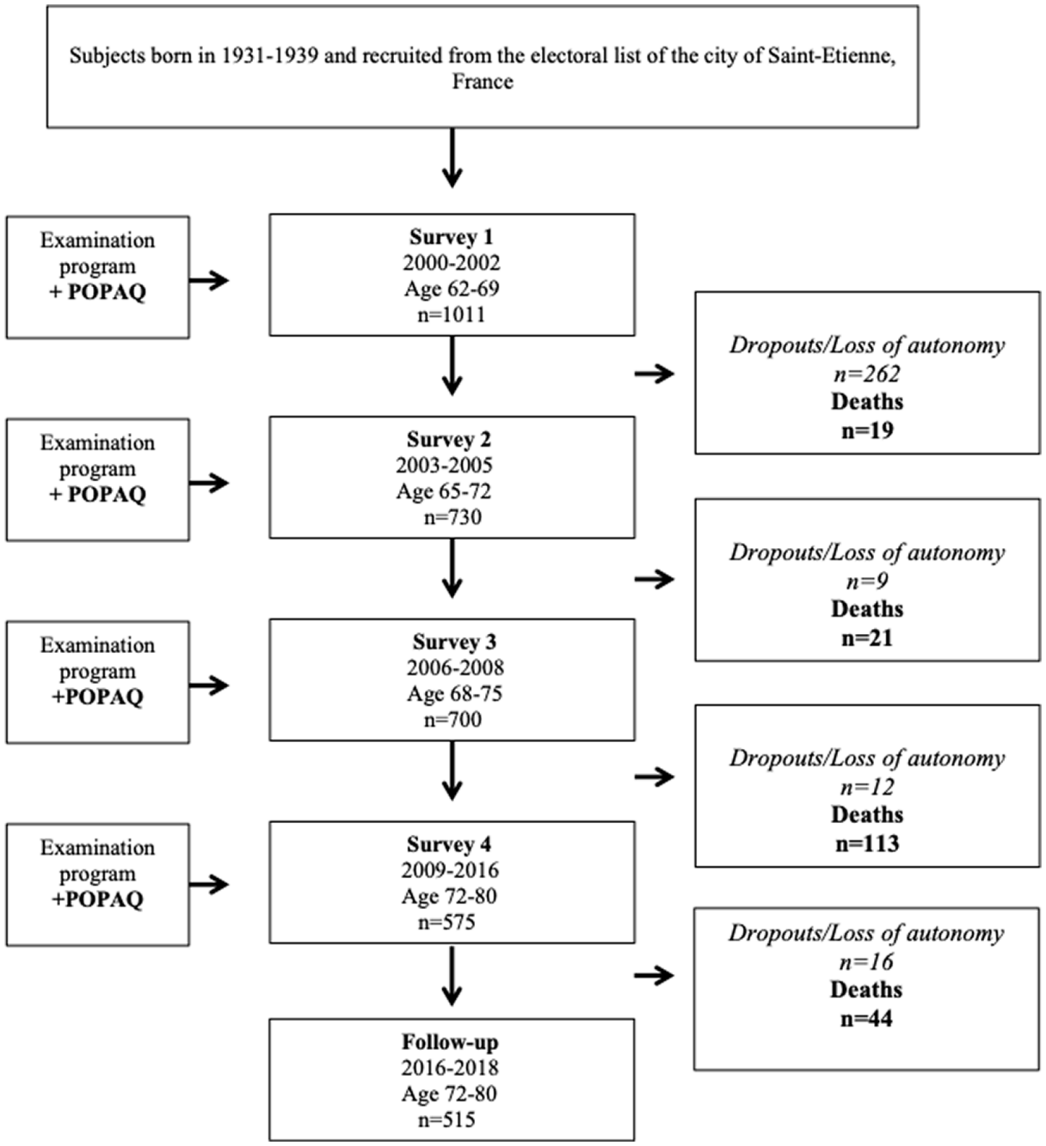
PROOF cohort study flowchart. POPAQ, population physical activity questionnaire.

From the 1011 65-year-old subjects initially included in 2001 (60% men), 515 subjects were followed until December 2019. One hundred and ninety-seven deaths (19.2%, including 77 CV deaths) and 195 CV events (19.3%) were reported over this 18-year follow-up. During the follow-up, there were 164 admissions to nursing homes (16.2%) and 135 dropouts (13.3%) (Figure 1).

The cohort was made up of 38.6% subjects with dyslipidemia, 36% with hypertension, 11% with family history of CV disease, 6% with diabetes mellitus and 8% were active smokers (Table 1). At baseline, there were 69% active subjects (*n* = 696), i.e., who reached MVPA recommendations (7.5 METs-h/week or 150 min/week of equivalent of brisk walking). Averages (standard deviation) of MVPA, LPA and SB were, respectively, 1.2 h/d (0.3), 5.8 h/d (1.1) and 6.6 h/d (2.3). The Spearman correlation coefficient between the 3 variables varied from 0.3 to 0.4, thus showing a slight correlation (*p* < 0.01).

**Table 1:** Characteristics of the population at baseline.

Variable (% or ± SD)	PROOF cohort (*n* = 1011)
Age (y)	65.6 (± 0.80)
Sex, females	609 (60)
BMI (kg.m^−2^)	25.3 ± 3.75
BMI < 25 kg.m^−2^	517 (51)
25 ≥ BMI < 30 kg/m^−2^	389 (39)
BMI ≥ 30 kg/m^−2^	105 (10)
Blood pressure (BP, min, max)	
Systolic BP, mmHg	140 (130, 155)
Diastolic BP, mmHg	90 (80, 93)
Cardiovascular risk factors	
None	195 (19)
1	359 (36)
2	304 (30)
≥3	153 (15)
Hypertension	365 (36)
hypercholesterolemia	381 (38)
Familial history	110 (11)
Type 2 diabetes	57 (6)
Smoking	
Current smoker	80 (8)
Former smoker	276 (28)
Non-smoker	648 (64)
Drug therapy	
Antihypertensive drug	223 (24)
Diuretics	103 (11)
ACE or ARB	92 (10)
Beta-blockers	86 (9)
Calcium channel blockers	69 (7)
Other antihypertensive drugs	16 (2)
Physical activity (h/d)	
LPA	5.8 (±1.1)
MVPA	1.2 (±0.3)
Sedentary behavior	6.6 (±2.3)
Biology	
Glycemia, g/l	1.01 (±0.21)
LDL cholesterol, g/l	1.54 (±0.32)
HDL cholesterol, g/l	0.52 (±0.14)
Total cholesterol, g/l	2.26 (±0.37)
Triglycerides, g/l	1.29 (±0.60)
C-reactive protein, mg/l (min, max)	2 (1.00, 3.90)

Mortality risk (all-cause deaths vs. non-death) was increased in men compared to women (52 vs. 48%, *p* < 0.0001) and in active smokers compared to previous and nonsmokers (8.3 vs. 4.3%, *p* = 0.02. *n* = 1.2 CV risk factors, *p* = 0.002).

Mortality risk (CV deaths vs. non-death) increased more in men than women (57 vs. 43%, *p* < 0.001), active smokers (10.4 vs. 4.2%, *p* = 0.01) and in those who had hypertension (71.4 vs. 56.2%, *p* = 0.01) and a higher CV risk (*n* = 1.6 vs. 1.1 CV risk factors, *p* < 0.001).

**Figure 2:**
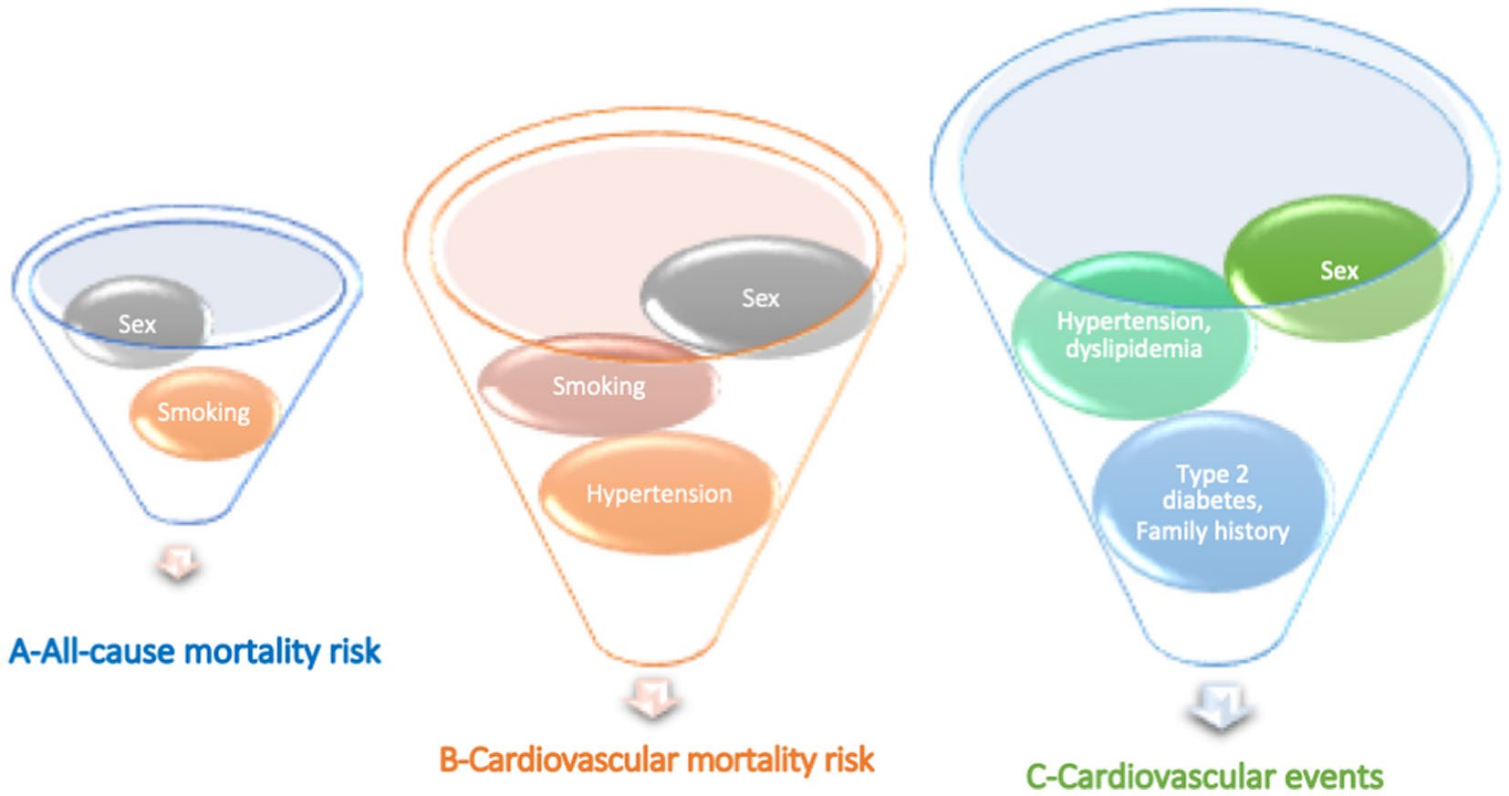
Main cardiovascular risks associated with **(A)** all-cause mortality risk, **(B)** cardiovascular mortality risk, and **(C)** cardiovascular events in the PROOF cohort.

CV events (vs. nonevent) were higher in women than in men (51 vs. 49%, *p* < 0.005), in those who had hypertension (73.3 vs. 50.6%, *p* < 0.001), dyslipidemia (49.2 vs. 36%, *p* = 0.001), type 2 diabetes (17.4 vs. 9.6%, *p* = 0.001), and with a family history of CV disease (14.9 vs. 9.9%, *p* = 0.04) (Figure 2).

**Figure 3:**
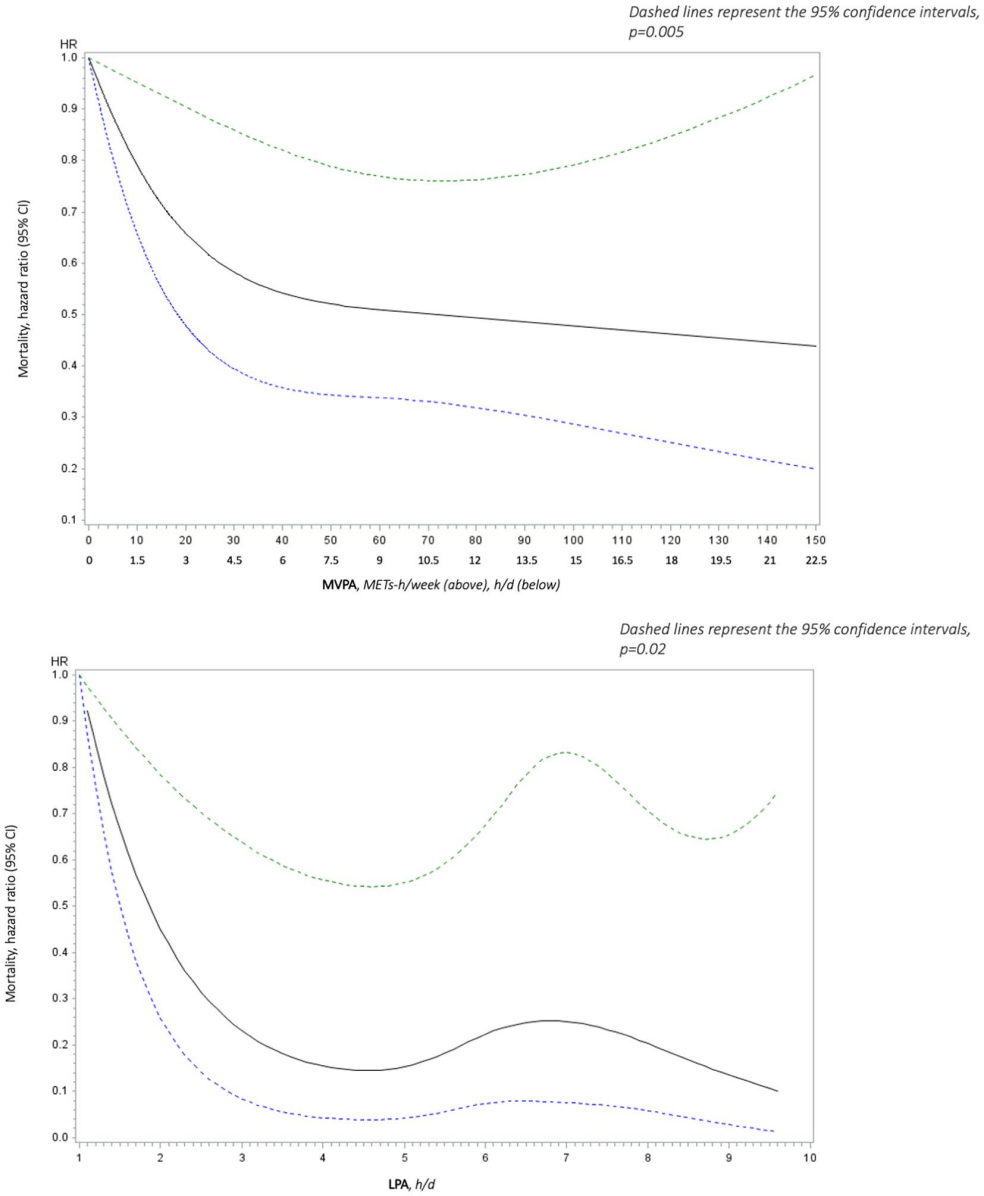
Adjusted (age, sex,) spline analyses of all-cause mortality hazard ratio (95% CI) as a function of light intensity physical activity (LPA in h/d) *above* and moderate to vigorous physical activity (MVPA in METs-h/week) *below*.

The relationship between the dose of PA or SB and the occurrence of mortality or CV event was assessed by cubic splines on COX survival models. Regarding all-cause mortality and CV mortality, the splines were significant for MVPA (*p* = 0.0007 and *p* = 0.0048, respectively), for LPA (*p* = 0.0401 and *p* = 0.0112, respectively), but not for SB (*p* = 0.2433 and *p* = 0.9075, respectively) (Figure 3 represents all-cause mortality). Reaching the MVPA guidelines reduced the hazard of mortality by about 13%.

Overall, mortality risk and CV events decreased with increasing LPA and MVPA (Figure 3), and this relationship was stronger in some conditions. Indeed, over 3 h of LPA (i.e., walking) per day led to a 81% [HR = 0.19 (95% CI 0.05 to 0.67) *p* < 0.01] and a 83% reduction in all-cause mortality in females and in those who had hypertension or type 2 diabetes [HR = 0.17 (95% CI 0.06 to 0.45) *p* = 0.0004], respectively. All-cause mortality was also 83% lower in active older adults (≥30 min/d of MVPA) who performed >3 h/d of LPA than in their active counterparts who did not perform it [HR = 0.17 (95% CI 0.06 to 0.52) *p* = 0.0017] (Supplementary Table S1).

This relationship was even stronger for CV mortality [HR = 0.21 (95% CI 0.07 to 0.67) *p* < 0.008, whereas HR of all-cause mortality was 0.46 (95% CI 0.23 to 0.95) *p* < 0.036] with over 3 h of LPA (Supplementary Table S2).

In general, mortality risk and CV events were increasing with the increase in SB, but without significant results.

**Figure 4:**
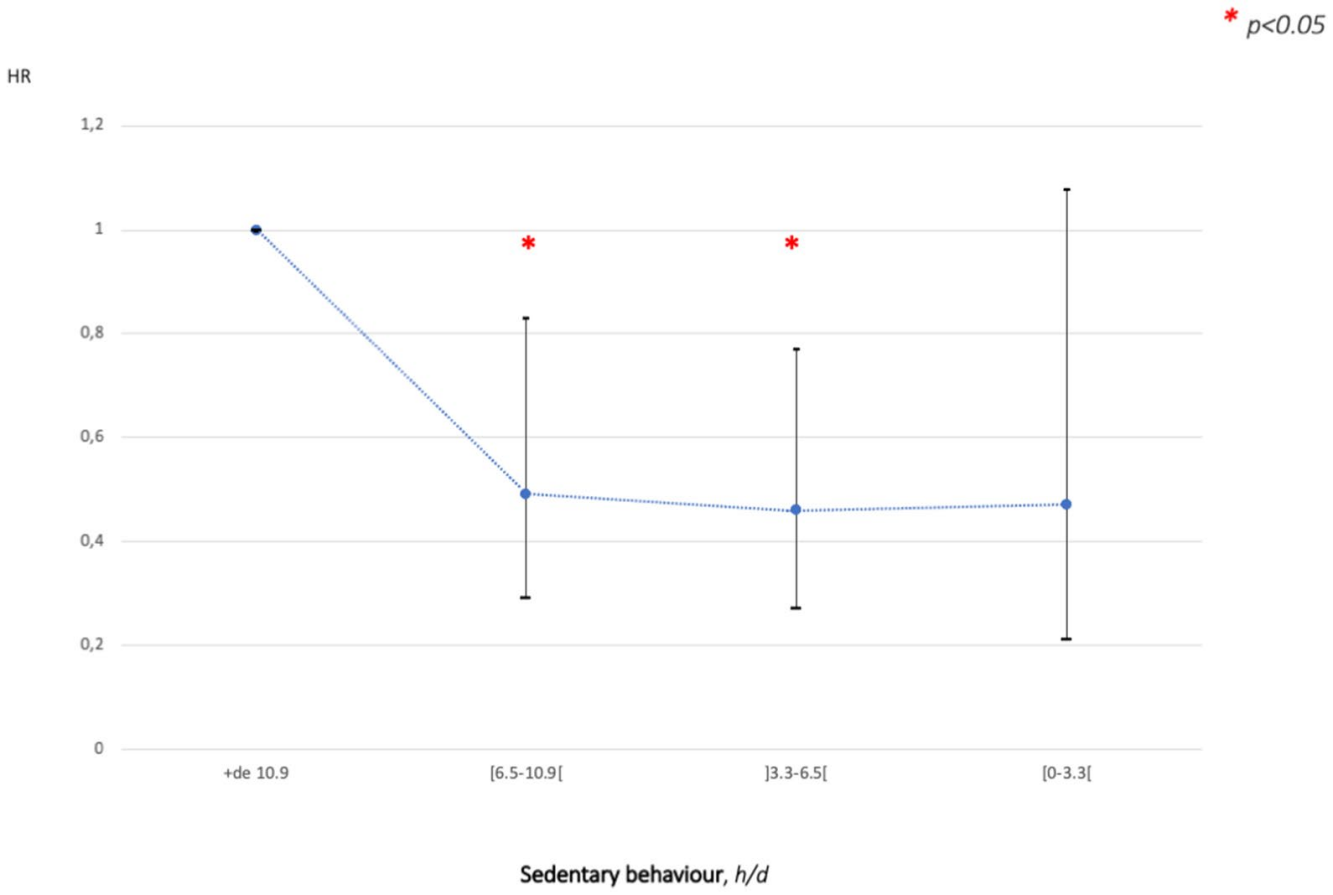
Adjusted (age, sex, CV risk factors: family history, hypertension, dyslipidaemia, type 2 diabetes, smoking, triglycerides and c-reactive protein) cardiovascular events hazard ratio (95% CI) as a function of sedentary behavior (h/d).

By using the nodes selected by the splines for MVPA, LPA and SB, new analyses were performed via COX models. Results were similar to the splines for MVPA and SB. In contrast, there was a significant reduction in CV events when SB was decreasing after adjustment for age, sex, CV risk factors (family history, hypertension, dyslipidemia, type 2 diabetes and smoking), triglycerides and CRP. Indeed, CV events in older people who decreased SB from 11 to 7 h/d were 51% lower [HR = 0.49 (95% CI 0.29 to 0.83)] and effects were confirmed up to 3.3 h/d [HR = 0.46 (95% CI 0.27 to 0.77)] (Figure 4).

## Discussion

This 18-year follow-up of the French prospective PROOF cohort study of older adults showed an inverse correlation between the doses of MVPA and LPA and mortality risk (either all-cause or CV). The magnitude of the association increased with increasing doses of MVPA and with LPA. This relationship was even stronger for CV mortality and for LPA only in some conditions (in males, in those who had hypertension or type 2 diabetes and in active people). Of note, the declines in mortality risk were high, even for low increases of LPA. The study also showed a positive association between SB and CV events, which decreased significantly even for low decreases of SB.

This prospective cohort study showed that a dose of MVPA below recommended level also reduced mortality among inactive adults aged ≥65 y (Figure 3). The older adults who performed <7.5 METs-h/week (<150 min/week) had up to a 12% reduction in mortality compared to those reporting 0 min/week of MVPA. These data are similar to that reported in a previous meta-analysis in a specific population aged >60 years old who did not reach yet 150 min of moderate-intensity PA per week (22% reduction in mortality with a dose-effect clearly identified) (1). As already reported, mortality was still reduced in those who engaged in a higher dose of MVPA: >20% for 15 METs-h/week and > 40% for 30 METs-h/week.

The reduction in all-cause mortality was also considerably strong for LPA. Subjects who reported only 1.5 h LPA per day had a 30% reduction in mortality risk. They had more than 80% reduction in mortality risk from 3 h LPA per day. These results were even stronger in women and in those who had chronic conditions (hypertension or type 2 diabetes). Similar findings were found for women in a previous meta-analysis (1).

This study suggested a curvilinear relationship between MVPA/LPA and mortality (all-cause and CV). There is a steep initial slope: the greatest benefits were seen in those who changed from doing the least or no MVPA/LPA to doing more. The shape of the dose-effect curve appeared to be stronger for LPA. Then the relationship was linear from a medium MVPA (i.e., 1.5 h/d)/LPA (i.e., 3 h/d) to a high dose of MVPA/LPA. Indeed, the increase in health benefits per unit increased in MVPA and LPA became smaller at the highest doses of activity; even if obvious, the greatest benefits concerned the highest doses of MVPA and the highest durations of LPA. Much of the strong inverse relationship between LPA and mortality was due to mortality from CVD. MVPA was less strongly related to CV mortality, but the decrease in risk was statistically significant.

Based on these results, we believe that the target for PA in the current recommendations (5–7, 18) might be not quite adapted and relevant for older adults and may discourage some of them. Our findings suggest that the dose-effect curve could be stronger for LPA. The decline in risk appears steeper at the lowest levels of LPA for older adults rather than for MVPA, and even the maximum benefits at the highest doses are higher. The fact that any effort could be worthwhile, e.g., just a walk at one’s own pace to start, may help convince the 31% of inactive subjects from the cohort and even 1.4 billion adults worldwide (27.5% of the world’s adult population) who do not practice any regular PA to become active (7). The current guidelines for PA have been widely broadcast (5–7, 18) but reaching MVPA intensities may be a goal too difficult for an older population that is reluctant or unable to engage in physical effort. Adapting these recommendations to older adults by underlining the dose-effect of regular LPA may encourage them to reduce SB and adopt or maintain a more active lifestyle.

### Strengths and limitations

To our knowledge, this is the first French cohort study to focus on the relationship between MVPA/LPA and SB and all-cause mortality in older adults. The main strengths of this study are the following: (1) the long follow-up of an older population-based cohort; (2) the rigorous follow-up of each subject of the cohort, using regular examination programs, various telephone solicitations to answer questionnaires and a very regular and rigorous collection of deaths and events; and (3) the relevant evaluation of MVPA, LPA and SB by questionnaire, validated with objective measurements (13, 15).

However, the present study has some limitations, mainly a bias in the analyses of the results could be due to some uncertainty in the collection of the data about MVPA, LPA and SB from the use of a self-reporting questionnaire. However, studies in which subjects reported their behaviors in questionnaires have historically provided the evidence that supports present global PA guidelines (19). Despite the technological evolution and the use of a wealth of objective measurement devices, an established standard for the measurement of PA does not exist due to the complexity of behaviors (20). Indeed, PA is multifaceted and concerns different domains (sport, leisure, occupation, transport, household), dimensions (frequency, duration, intensity and type), determinants (21, 22) and still more (23). Therefore, any measurement is relevant and depends on the studied facets of PA, study aims, sample population and more (24).

### Implications for policy and practice

Epidemiological studies are a powerful tool for scientific societies to promote recommendations regarding PA intended for the general population (25). To date, WHO recommendations for older adults are not different from those for middle-aged adults (5–7, 18). The only mention is: “as part of their weekly physical activity, older adults should do varied multicomponent physical activity that emphasizes functional balance and strength training at moderate or greater intensity, on 3 or more days a week, to enhance functional capacity and to prevent falls.” Prevention of falls is also a relevant target in PA promotion (26, 27). The reduction of SB must be associated with the promotion of LPA in the public health messages for older adults.

Our cohort study showed that an equivalent daily dose of walking at low intensity (at least 1.5 h at one’s own pace) has already a beneficial effect on health in older adults with a 30% reduction in mortality risk. This activity could be repeated in several bouts of some minutes to reach the set objective. These findings are in accordance with the reduction in CV events observed after reducing SB by at least 3 h/d.

Ninety more minutes of accumulated low intensity walking per day associated with 90 less minutes of sitting time per day could be a reasonable target dose in subjects over 65. A modification of the recommendations for PA in older adults that emphasizes the health benefits of more LPA and less SB periods may thus be warranted and beneficial. The prescription of PA aims to lead older adults with an intention to change, to accompany them in this change, and to encourage them to maintain a more active lifestyle in the long term. This prescription, because it affects the most susceptible, would also reduce social inequalities in health (26). The widespread diffusion of this message will encourage more older adults to include more LPA and less SB periods in their usual daily activities, without experiencing high levels of fatigue or pain. This message should be relayed by any healthcare professional who plays a key and essential role in promoting PA and reducing SBs in older adults.

Finally, even if the intensity of PA (LPA and MVPA) required to decrease mortality and improve quality of life remains a matter of debate, it could be summarized as ‘Even a little light-intensity is good, more moderate-intensity may be better!’ (28). This message provides reassurance that engaging in PA is worthwhile, even in very old age.

To convey a simple and attractive message, we thus recommend at least 1.5 more hours of LPA per day as a first target for older adults. This could include leisure time PA or daily life activities or an equivalent of leisure walking.

Scientific evidence is now emerging to show that there may be health benefits from LPA (1.6–2.9 METs), and from replacing SB (<1.6 METs) with LPA, while the dose of MVPA (≥3 METs) is held constant (8, 29–32).

## Conclusion

The PROOF cohort study shows a clear dose–response between the dose of LPA, MVPA, SB and risk of mortality and even risk of CVDs. This study corroborates that PA represents a continuum with a LPA component which appears to have a potent effect on health per unit of time. LPA engagement may provide additional health benefits, which is independent of MVPA. LPA offers another pathway to replace SB and to accumulate daily energy expenditure, especially for older people, because LPA does not require a high level of starting fitness or planification. It usually involves only incidental daily living and increased movement during leisure time. These findings provide additional evidence to support the inclusion of LPA in future PA guidelines.

## Data availability statement

The raw data supporting the conclusions of this article will be made available by the authors, without undue reservation.

## Ethics statement

The studies involving human participants were reviewed and approved by the Ethics Committee of Saint-Etienne University Hospital. The patients/participants provided their written informed consent to participate in this study.

## Author contributions

DH had full access to all data in the study and takes responsibility for the integrity of the data and the accuracy of the data analysis. CD, MBe, VP, SC, FR, J-CB, and DH had the idea for and designed the study and developed the methodological study design. CD, MBr, and DH conducted the literature search, responsible for interpretation of data, drafted the manuscript, and submitted the manuscript for publication. MBr and DH were responsible for collection and analyses of data. CD provided statistical expertise. CD, MBr, MBe, VP, SC, MG, HF, NB, JG, BB, J-CB, FR, and DH critically revised the manuscript for important intellectual content. All authors made a substantial contribution to the conception and design of the study, read, and approved the final version of the manuscript.

## Funding

This PROOF study was funded through three grants from the French Ministry of Health (Programmes Hospitaliers de Recherche Clinique: PHRC National PROOF 1998; PHRC National SYNAPSE 2002; and PHRC Regional Telamons 2003).

## Conflict of interest

The authors declare that the research was conducted in the absence of any commercial or financial relationships that could be construed as a potential conflict of interest.

## Publisher’s note

All claims expressed in this article are solely those of the authors and do not necessarily represent those of their affiliated organizations, or those of the publisher, the editors and the reviewers. Any product that may be evaluated in this article, or claim that may be made by its manufacturer, is not guaranteed or endorsed by the publisher.

## Footnotes

^1^ https://sites.google.com/site/compendiumofphysicalactivities/home
