# Supplementary material for: Relationship between moderate-to-vigorous, light intensity physical activity and sedentary behavior in a prospective cohort of older French adults: a 18-year follow-up of mortality and cardiovascular events ─ the PROOF cohort study
Source: Front Public Health. 2023 Jun 7;11:1182552. doi: 10.3389/fpubh.2023.1182552 (PMC10284080; doi:10.3389/fpubh.2023.1182552)
Supplement: Supplementary file 1 [file Table_1.DOCX]

**Supplementary Tables 1.** Association between moderate-to-vigorous intensity physical activity (MVPA in METs-h/wk), light intensity physical activity (LPA in h/d), sedentary behavioral (SB in h/d) and all-cause mortality hazard ratio (95% CI) (*adjusted for age, sex, family history, hypertension, dyslipidemia, type 2 diabetes, smoking, triglycerides and CRP*) (A)-in the PROOF cohort, (B)-in female, (C)-in those who have hypertension or type 2 diabetes, (D)-in active subjects (MVPA ≥ 0.5 h/d).

A-

B-

C-

D-

*MVPA: moderate-to-vigorous intensity physical activity (METs-h/wk), LPA: light intensity physical activity (h/d),*

*SB: sedentary behaviour (h/d)*

**Supplementary Table 2.** Association between moderate-to-vigorous intensity physical activity (MVPA in METs-h/wk), light intensity physical activity (LPA in h/d), sedentary behavioral (SB in h/d) and cardiovascular mortality hazard ratio (95% CI) (*adjusted for age, sex, hypertension, dyslipidemia, type 2 diabetes, smoking, triglycerides and CRP*) in the PROOF cohort.

*MVPA: moderate-to-vigorous intensity physical activity (METs-h/wk), LPA: light intensity physical activity (h/d), SB: sedentary behaviour (h/d)*
